# Efficacy and safety of a novel polytetrafluoroethylene‐coated self‐expandable metal stent for distal malignant biliary obstruction

**DOI:** 10.1002/deo2.70010

**Published:** 2024-09-03

**Authors:** Hiroki Nakagawa, Tsuyoshi Takeda, Takeshi Okamoto, Tatsuki Hirai, Takafumi Mie, Takaaki Furukawa, Akiyoshi Kasuga, Takashi Sasaki, Masato Ozaka, Takahisa Matsuda, Yoshinori Igarashi, Naoki Sasahira

**Affiliations:** ^1^ Department of Hepato‐Biliary‐Pancreatic Medicine Cancer Institute Hospital of Japanese Foundation for Cancer Research Tokyo Japan; ^2^ Department of Internal Medicine Omori Medical Center Division of Gastroenterology and Hepatology Toho University Tokyo Japan

**Keywords:** adverse event, distal malignant biliary obstruction, polytetrafluoroethylene, recurrent biliary obstruction, self‐expandable metal stent

## Abstract

**Background:**

Stent migration and sludge formation remain significant problems associated with covered self‐expandable metal stents (CSEMSs). The EGIS biliary stent fully covered flare type (EGIS biliary stent), a new type of polytetrafluoroethylene‐coated self‐expandable metal stent with low axial force and an anti‐migration system, was developed to overcome these disadvantages. We conducted this study to evaluate the efficacy and safety of this stent in comparison with conventional CSEMS (c‐CSEMS).

**Methods:**

We retrospectively analyzed consecutive patients with unresectable pancreatic cancer who received initial CSEMS for distal malignant biliary obstruction. The primary outcome was time to recurrent biliary obstruction (RBO). Secondary outcomes included technical success rate, functional success rate, stent‐related adverse events, causes of RBO, and re‐intervention.

**Results:**

A total of 40 patients were included (EGIS group: 20; c‐CSEMS group: 20). The technical and functional success rates were similar between the two groups. Stent‐related adverse event rates (20% vs. 15%, *p* > 0.99) and overall RBO rates (56% vs. 50%, *p* > 0.99) were not significantly different between the two groups. Stent migration was the most common cause of RBO in the EGIS group, while stent occlusion was in the c‐CSEMS group. The median time to RBO (102 vs. 434 days, *p* = 0.10) was not significantly different between the two groups. Endoscopic transpapillary re‐intervention was successful in most patients in both groups.

**Conclusions:**

The EGIS biliary stent was not associated with a longer time to RBO compared to c‐CSEMS. Further improvements, especially against stent migration, are needed to improve its efficacy.

## INTRODUCTION

Endoscopic placement of self‐expandable metal stents (SEMSs) is recommended for the treatment of unresectable distal malignant biliary obstruction (MBO).^1^, ^2^ SEMSs are classified into uncovered SEMSs (USEMSs) and covered SEMSs (CSEMSs). Disadvantages of USEMSs include their high occlusion rate due to tumor ingrowth and inability to be removed.^3^ CSEMSs, which were designed to overcome these disadvantages, have been reported to be superior to USEMSs, especially in patients with pancreatic cancer.^4^ On the other hand, recurrent biliary obstruction (RBO) due to stent migration and sludge formation remain significant problems associated with CSEMSs. Stent‐related adverse events (AEs) including pancreatitis and cholecystitis are also major concerns.^5^ ^6^ As SEMSs with high axial force (AF) are considered risk factors for pancreatitis^7^ and cholecystitis,^8^ CSEMSs with low AF and an anti‐migration system may be the ideal SEMS for distal MBO.^9^

The EGIS biliary stent fully covered flare type (EGIS biliary stent), is a new type of polytetrafluoroethylene (PTFE)‐coated SEMS with low AF and an anti‐migration system. PTFE‐coated stents are reported to have several advantages including resistance to bacterial growth, high durability due to resistance to chemicals and heat, and low coefficient of friction,^10^ which may reduce sludge formation.^11^ The low AF was anticipated to reduce stent‐related AEs and the flared proximal end was designed to reduce stent migration. In summary, the EGIS biliary stent was designed to prolong the time to RBO (TRBO) by preventing sludge formation and stent migration and reducing stent‐related AEs through low AF.

We conducted this retrospective study to evaluate the efficacy and safety of this new type of SEMS in comparison with conventional CSEMS (c‐CSEMS).

## METHODS

### Patients

Consecutive patients with unresectable pancreatic cancer who received initial CSEMS for the palliation of distal MBO between March 2021 and October 2022 were identified from our prospectively maintained database. Excluded patients were as follows: (1) patients who received a CSEMS above the papilla, (2) patients who received an anti‐reflux metal stent, (3) patients with a concomitant hilar biliary obstruction, and (4) patients with surgically altered anatomy. Types of CSEMS used were mainly decided based on the time period during which the patient underwent CSEMS placement. The EGIS biliary stent was generally used between January 2022 and October 2022, while c‐CSEMS was used between March 2021 and January 2022. This study was conducted in accordance with a master protocol (2023‐GB‐077) for gastrointestinal, hepatic, and pancreatobiliary diseases, which was approved by the institutional review board of our institution. Informed consent for this study was waived due to its retrospective design. The study was publicized on the hospital website, allowing patients to opt out of this study for any reason.

### Design of SEMS

**FIGURE 1:**
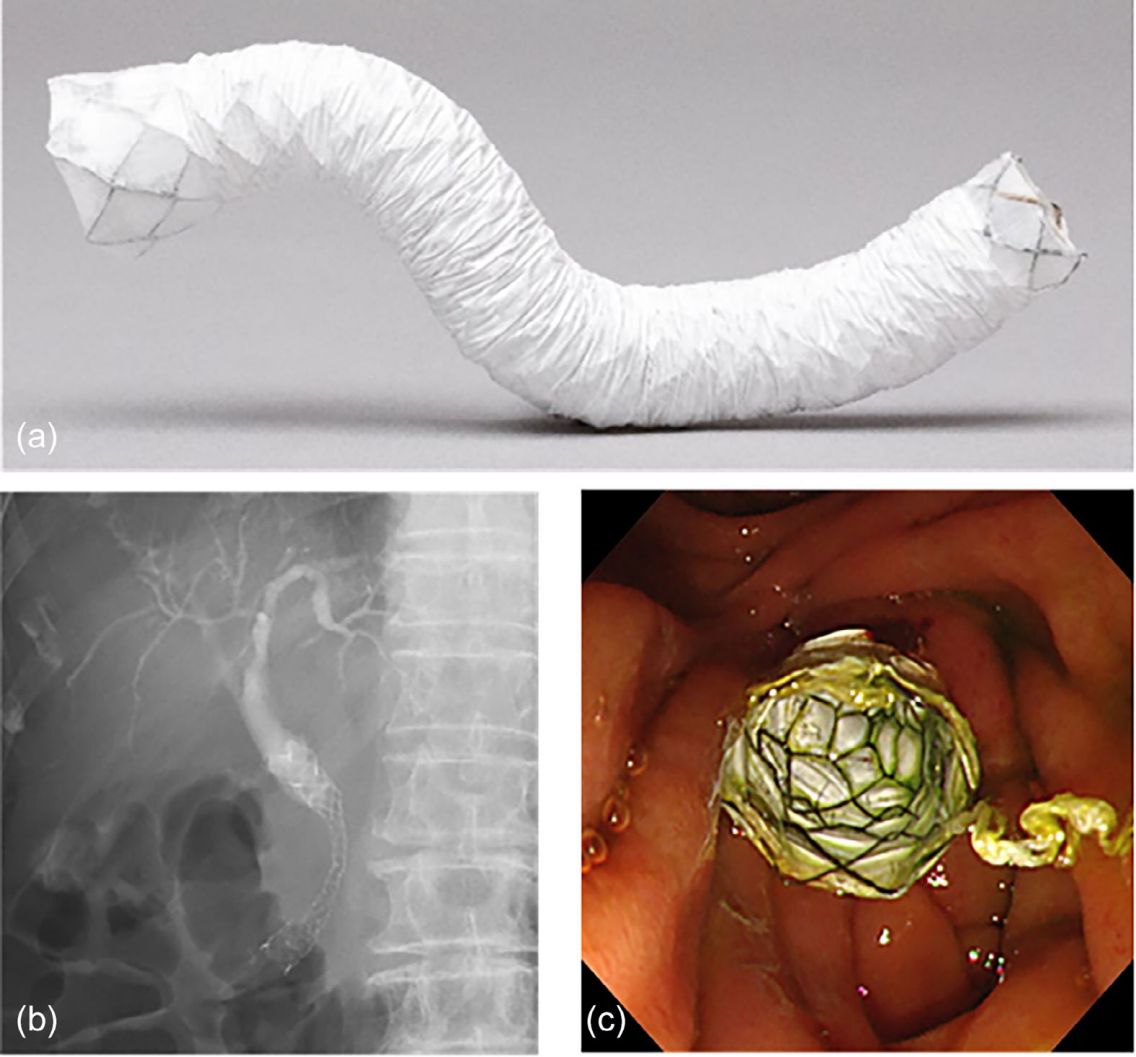
EGIS biliary stent fully covered flare type. (a) This stent has a low axial force and conformability to the shape of the bile duct. (b) Fluoroscopic view of the stent. (c) Endoscopic view of the stent.

The EGIS biliary stent (SB‐Kawasumi Laboratories Inc.) is a hook and cross‐type fully covered SEMS with a flared proximal end (Figure 1). The stent is made of nitinol wire and the wire is highly polished to prevent biliary sludge formation. The composition ratio of the hook and cross wires of the stent is 5:1, which makes the AF very low. In addition, the stent is coated with PTFE, which is considered to have higher durability compared to conventional silicone. The proximal end of the stent is flared with a 16‐mm diameter to prevent outward migration. A lasso is attached to the lower end of the stent to allow for adjustment of the stent position after placement. Radio‐opaque markers are placed at the proximal end, distal end, and flare of the stent to facilitate fluoroscopic visualization. The diameter of the stent is 10 mm, and the lengths of the stent are 6 and 8 cm. The diameter of the delivery system is 8 Fr.

The c‐CSEMS used in this study was HANAROSTENT Biliary Full Cover NEO (M.I. Tech). Stents with diameters of 10 mm and lengths of 6, 7, and 8 cm were used in this study.

### Endoscopic Procedures

Endoscopic retrograde cholangiopancreatography (ERCP) was performed using a therapeutic duodenoscope (JF‐260V, or TJF‐Q290V; Olympus Medical Systems, Tokyo, Japan) under conscious sedation with intravenous midazolam and pethidine. Endoscopic sphincterotomy (EST) with a small to moderate‐size incision was performed before stent placement. The length of the SEMS used was determined based on cholangiographic findings. All ERCP procedures were performed by expert endoscopists with more than five years of experience in therapeutic endoscopy or trainees under their direct supervision. Prophylactic rectal nonsteroidal anti‐inflammatory drugs (NSAIDs) were used at the endoscopist's discretion.

### Outcome Measurements and Definitions

Outcomes of SEMS placement were basically evaluated according to Tokyo Criteria 2014.^12^ The primary outcome was TRBO. TRBO was defined as the period between stent placement and RBO. RBO was defined as a composite endpoint of either stent occlusion or stent migration, which was evaluated for those who achieved technical and functional success after CSEMS placement. Secondary outcomes included technical success rate, functional success rate, stent‐related AEs, causes of RBO, and re‐intervention. Technical success was defined as successful stent placement in the intended location. Functional success was defined as a 50% decrease in, or normalization of, serum bilirubin level within 14 days after stent placement. For patients who underwent endoscopic nasobiliary drainage or percutaneous transhepatic biliary drainage before SEMS placement, functional success was achieved if the serum bilirubin level was not exacerbated after the procedure. The severity of AEs was graded according to the American Society of Gastrointestinal Endoscopy lexicon guidelines.^13^ Tumor involvement of the pancreatic duct was defined as main pancreatic duct obstruction with upstream pancreatic duct dilation. Tumor involvement of the orifice of the cystic duct (OCD) was defined as tumor extension around OCD shown by computed tomography. The diameter of the common bile duct was defined as the maximum diameter measured by cholangiography at the time of SEMS placement. Follow‐up data were confirmed until August 31, 2023.

### Statistical Analysis

Continuous variables are expressed as median (interquartile range) and were compared using the Mann‐Whitney U test. Categorical variables are expressed as absolute numbers (proportions) and were analyzed using either the chi‐square test or Fisher's exact test as appropriate. Overall survival was defined as the time from CSEMS placement to death or the last follow‐up. Overall survival and TRBO were evaluated using the Kaplan‐Meier method and were compared using the log‐rank test. *p*‐Values < 0.05 were considered statistically significant. Statistical analyses were performed using EZR software version 1.54.^14^

## RESULTS

### Patient characteristics

**FIGURE 2:**
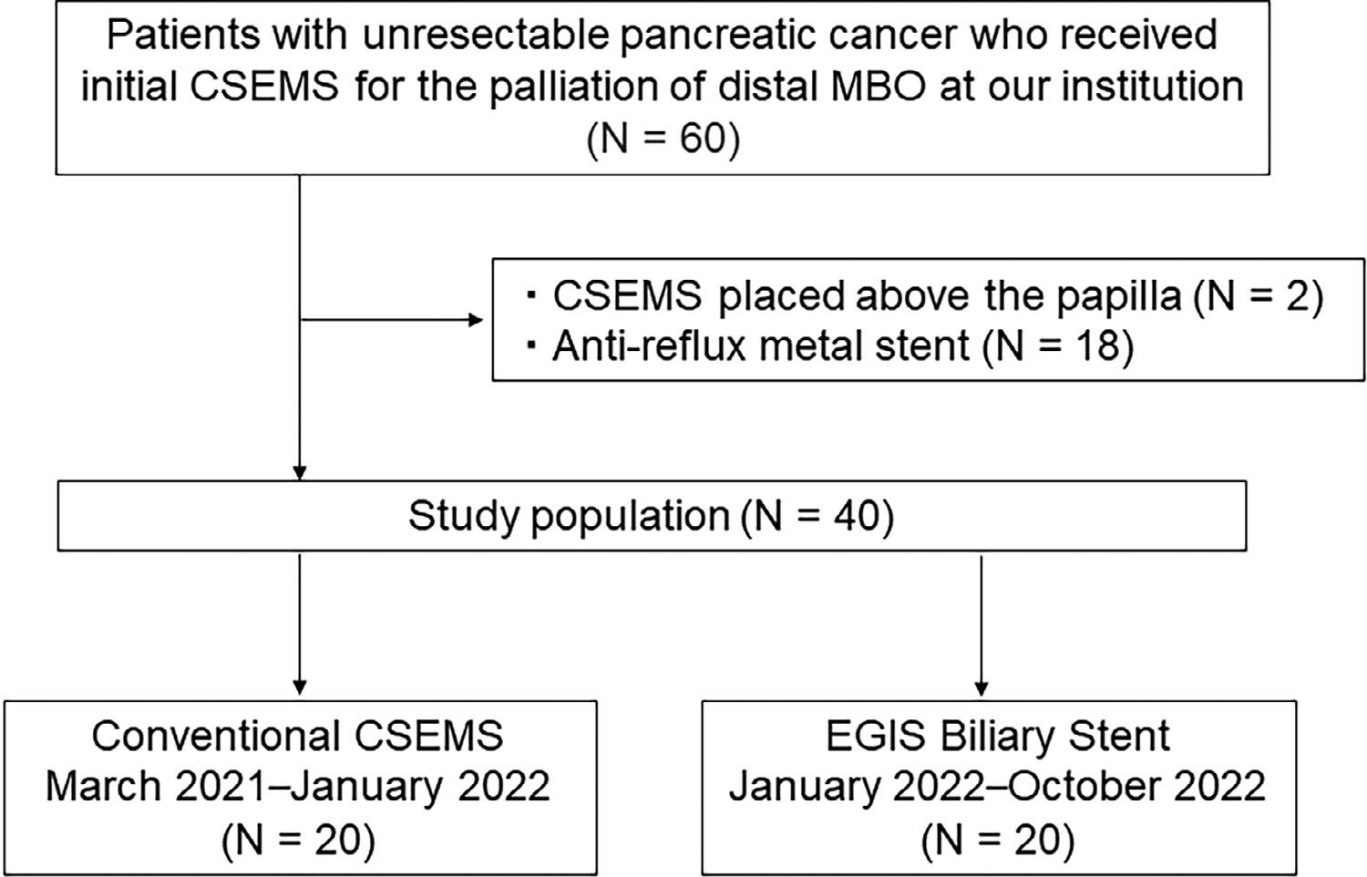
Patient flowchart. CSEMS, covered self‐expandable metal stent; MBO, malignant biliary obstruction.

A total of 40 patients were included in this study (Figure 2). Twenty patients received EGIS biliary stent (EGIS group) and 20 received c‐CSEMS (c‐CSEMS group). Patient characteristics including median follow‐up period (216 vs. 318 days, *p* = 0.19) were not significantly different between the two groups (Table 1).

**TABLE 1:** Baseline characteristics between the two groups.

	EGIS *N* = 20	c‐CSEMS *N* = 20	*p*‐value
Age, years	67.5 (65.0–74.3)	68.0 (58.3–74.5)	0.63
Sex, male	12 (60%)	10 (50%)	0.75
ECOG performance status, 0/1/2/3	15/5/0/0	11/6/2/1	0.37
Tumor status			>0.99
‐ Locally‐advanced	7 (35%)	7 (35%)	
‐ Metastatic	13 (65%)	13 (65%)	
Post‐cholecystectomy	2 (10%)	0 (0%)	0.49
Tumor involvement of OCD	3 (15%)	1 (5%)	0.61
Diameter of the common bile duct, mm	10.7 (8.9–15.0)	9.0 (7.9–11.1)	0.22
Tumor involvement of the pancreatic duct	18 (90%)	18 (90%)	>0.99
Treatment for cancer after stent placement			0.33
‐ Chemotherapy	13 (65%)	17 (85%)	
‐ Chemoradiation therapy	1 (5%)	1 (5%)	
‐ Best supportive care	6 (30%)	2 (10%)	
Follow‐up period, days	216 (63–475)	318 (136–506)	0.19

Continuous variables are expressed as median (interquartile range) and categorical variables are expressed as absolute numbers (proportions). Abbreviations: c‐CSEMS, conventional covered self‐expandable metal stent; ECOG, Eastern Cooperative Oncology Group; OCD, orifice of the cystic duct.

Procedural characteristics are shown in Table 2. Stents with 8 cm in length were most commonly used in the EGIS group, while stents with 7 cm in length were used in the c‐CSEMS group. EST was performed in all patients. Rate of stents placed across the OCD and prophylactic rectal NSAID use were not significantly different between the two groups.

**TABLE 2:** Procedural characteristics between the two groups.

	EGIS *N* = 20	c‐CSEMS *N* = 20	*p*‐value
Stent length			<0.001
‐ 6 cm	4 (20%)	8 (40%)	
‐ 7 cm	Not available	9 (45%)	
‐ 8 cm	16 (80%)	3 (15%)	
Endoscopic sphincterotomy	20 (100%)	20 (100%)	>0.99
Pancreatic stent placement	0 (0%)	0 (0%)	>0.99
Stent placed across the OCD	17 (85%)	17 (85%)	>0.99
Prophylactic rectal NSAIDs use	14 (70%)	12 (60%)	0.74

Categorical variables are expressed as absolute numbers (proportions). Abbreviations: c‐CSEMS, conventional covered self‐expandable metal stent; N/A, not available; OCD, orifice of the cystic duct; NSAIDs, nonsteroidal anti‐inflammatory drugs.

### Outcome measures

The outcomes of SEMS placement are shown in Table 3. The technical and functional success rates were high in both groups. The reasons for functional failure were exacerbation of bilirubin levels before achieving functional success in three patients (EGIS group: 2; c‐CSEMS group: 1) and death due to worsening of primary cancer in one patient (c‐CSEMS 1). Two patients in the EGIS group developed cholangitis (due to non‐occlusion cholangitis and food impaction, respectively) and received endoscopic nasobiliary drainage. One patient received an anti‐reflux metal stent after cholangitis resolved, while the other patient emergently received a plastic stent because the patient accidentally self‐removed the endoscopic nasobiliary drainage tube. The overall stent‐related AE rates were not significantly different between the two groups (20% vs. 15%, *p* > 0.99). Cholecystitis occurred as early AEs in two patients (occurring 18 days and 24 days after stent placement in the EGIS and c‐CSEMS groups, respectively) and as late AEs in the remaining two patients (both occurring 38 days after stent placement in the EGIS group). Tumor involvement of OCD was present in two of the four patients. Percutaneous transhepatic gallbladder drainage was performed in all four patients. Pancreatitis occurred in two patients in the c‐CSEMS group. Pancreatitis resolved after conservative treatment in both patients (one patient underwent stent removal). Transcatheter arterial embolization was performed for bleeding due to pseudoaneurysm, which was developed 25 days after SEMS placement in the EGIS group.

**TABLE 3:** Clinical outcomes between the two groups.

	EGIS *N* = 20	c‐CSEMS *N* = 20	*p*‐value
Technical success	20 (100%)	20 (100%)	>0.99
Functional success	18 (90%)	18 (90%)	>0.99
Stent‐related adverse events	4 (20%)	3 (15%)	>0.99
Cholecystitis	3 (15%)	1 (5%)	0.61
Mild/moderate/severe	0/2/1	0/1/0	
Pancreatitis	0 (0%)	2 (10%)	0.49
Mild/moderate/severe		1/1/0	
Bleeding	1 (5%)	0 (0%)	>0.99
Mild/moderate/severe	0/0/1		
Recurrent biliary obstruction^a^	10 (56%)	9 (50%)	>0.99
Stent occlusion	3 (17%)	6 (33%)	0.44
Biliary sludge	2	6	
Kinking	1	0	
Stent migration	7 (39%)	2 (11%)	0.12
Inward	2	1	
Outward	5	1	
Complete/incomplete	4/1	1/0	
Non‐occlusion cholangitis	0 (0%)	1 (6%)	>0.99

Categorical variables are expressed as absolute numbers (proportions). ^a^Denominators adjusted to exclude four patients who did not achieve functional success (two patients in each group). Abbreviation: c‐CSEMS, conventional covered self‐expandable metal stent.

**FIGURE 3:**
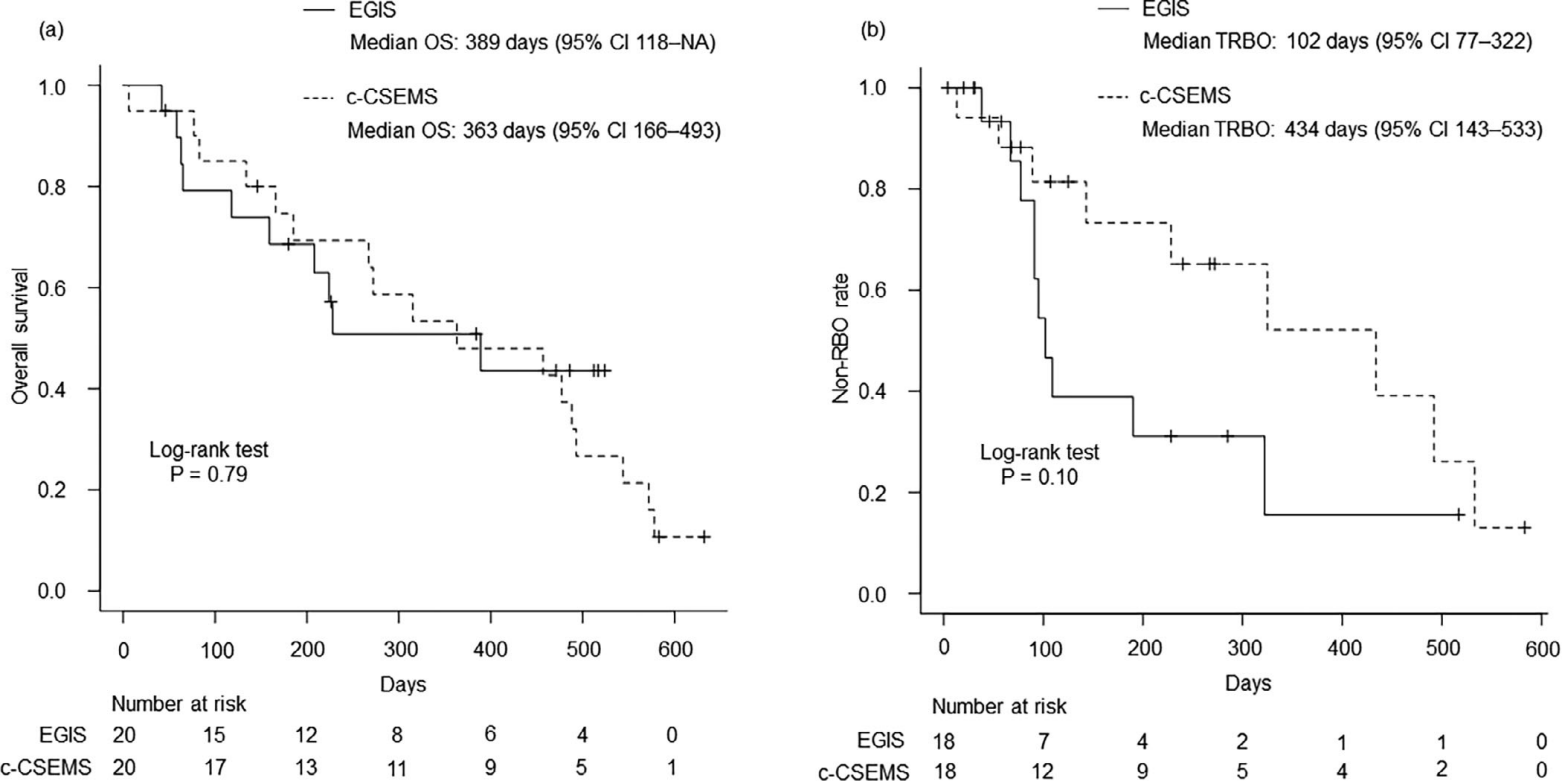
Kaplan–Meier curves by stent group. (a) Overall survival. (b) Time to recurrent biliary obstruction. OS, overall survival; CI, confidence interval; NA, not applicable; c‐CSEMS, conventional covered self‐expandable metal stent; RBO, recurrent biliary obstruction; TRBO, time to recurrent biliary obstruction.

The overall RBO rates were not significantly different between the two groups (56% vs. 50%, *p* > 0.99). Stent migration was the most common cause of RBO in the EGIS group, while stent occlusion was in the c‐CSEMS group. Kaplan‐Meier curves of overall survival and TRBO are illustrated in Figure 3. Median overall survival was similar between the two groups (389 vs. 363 days, *p* = 0.79), while median TRBO was shorter in the EGIS group (102 vs. 434 days, *p* = 0.10), although the difference was not statistically significant.

Re‐intervention was performed in 10 and nine patients in the EGIS and c‐CSEMS groups, respectively. Stent removal was successful in all attempted cases in both groups (EGIS group: six; c‐CSEMS group: seven), while stent removal was not attempted in one patient in the c‐CSEMS group due to the endoscopist's discretion. Endoscopic transpapillary re‐intervention was successful in eight patients in the EGIS group; six patients underwent SEMS placement, one underwent plastic stent placement, and one underwent SEMS and plastic stent placement for distal and hilar biliary obstruction. Of the two patients in which transpapillary re‐intervention could not be performed due to difficulty in cannulating the bile duct, one patient underwent percutaneous biliary drainage and one underwent endoscopic ultrasound‐guided hepaticogastrostomy. On the other hand, endoscopic transpapillary re‐intervention was successful in all nine patients in the c‐CSEMS group; four patients underwent SEMS placement, three underwent plastic stent placement, one underwent temporary endoscopic nasobiliary drainage and removal of biliary sludge, and one patient with duodenal stricture underwent endoscopic ultrasound‐guided hepaticogastrostomy following endoscopic nasobiliary drainage.

## DISCUSSION

In the present study, we evaluated the efficacy and safety of a newly designed PTFE‐coated SEMS with low AF in comparison with c‐CSEMS. The EGIS biliary stent was not associated with longer TRBO compared to c‐CSEMS (102 days vs. 434 days, *p* = 0.10). Stent migration was the most common cause of RBO in the EGIS group, while stent occlusion was in the c‐CSEMS group. Other outcomes including technical and functional success rates, stent‐related AEs (20% vs. 15%, *p* > 0.99), and overall RBO rates (56% vs. 50%, *p* > 0.99) were also not significantly different between the two groups.

PTFE‐coated SEMSs, which are designed to have properties including resistance to chemicals and heat and low coefficient of friction,^10^, ^15^, ^16^ have been developed to prolong TRBO through inhibition of sludge formation. The first reported PTFE‐coated SEMS was VIABIL Biliary Endoprosthesis (W.L. Gore & Associates), which could be deployed percutaneously.^15^, ^17^ This stent consisted of an inner PTFE membrane and an outer helical nitinol wire and was reported to have a significantly prolonged stent patency compared to USEMSs by preventing tumor ingrowth.^17^ The second reported PTFE‐coated SEMS was a ComVi stent (Taewoong Medical Device), which could be inserted endoscopically.^18^ This stent consisted of a PTFE membrane sandwiched between two layers of wire with low AF. Isayama et al.^18^ compared this stent with a non‐PTFE‐coated SEMS and reported that stent patency was not significantly different between the two stents, albeit with a significantly lower migration rate (2.1% vs. 17.0%, *p* = 0.030).

Recently, several novel PTFE‐coated SEMSs have been developed. One is the HILZO Biliary covered stent (BCM Co., Ltd.). This stent is coated with two PTFE membranes and has dumbbell‐shaped flared ends. The inner PTFE membrane is designed to prevent biliary sludge and food impaction, while the outer membrane is designed to prevent tumor ingrowth. The dumbbell‐shaped flared ends were designed to reduce sludge formation and stent migration. Miyazawa et al.^11^ compared this stent with other types of CSEMSs and reported that this stent had lower RBO rates (HILZO vs. partially covered SEMS: 22.2% vs. 50.0%, *p* = 0.033; HILZO vs. fully covered SEMS: 22.2% vs. 50.0%, *p* = 0.023) and longer TRBO (HILZO vs. partially covered SEMS: not reached vs. 290 days, *p* = 0.074; HILZO vs. fully covered SEMS: not reached vs. 323 days, *p* = 0.049) compared to other types of CSEMSs. Finally, the EGIS biliary stent is a new PTFE‐coated SEMS with low AF and an anti‐migration system. This stent was expected to prolong TRBO by preventing sludge formation and stent migration. However, in this study, RBO due to sludge formation and stent migration was higher than expected. The high rate of sludge formation may be attributable to a lack of PTFE coating on the inner side of the stent. Although low radial force is considered a risk factor for stent migration,^19^ radial force of the EGIS biliary stent is believed to be similar to other stents. Minaga et al.^20^ reported that three stent flare structure variables (outer diameter of the flare, height of the flare, and taper angle of the flare) may strongly affect the anti‐migration property of the stent when the stent is fully expanded. Therefore, the high rate of stent migration may be due to the low coefficient of friction of PTFE^10^ and the insufficient function of the anti‐migration system resulting from the stent flare structure. The dumbbell‐shaped flared ends of the HILZO Biliary covered stent may be more effective for preventing stent migration.

With respect to stent‐related AEs, the incidence of pancreatitis and cholecystitis after SEMS placement has been reported to be 6.0%–9.3%^7^, ^21^, ^22^ and 6.9%–7.4%,^6^, ^8^ respectively. None of the patients developed pancreatitis in the EGIS group, while two patients (10%) developed pancreatitis in the c‐CSEMS group. Risk factors for pancreatitis include SEMS with high AF, non‐pancreatic cancer,^7^ absence of tumor involvement of pancreatic duct,^23^ and absence of pancreatic duct dilation.^22^ As the rate of tumor involvement in the pancreatic duct was similar between the two groups, the low AF of the EGIS biliary stent may explain the absence of pancreatitis after stent placement in this study. Cholecystitis occurred in three and one patient in the EGIS and c‐CSEMS groups, respectively (15% vs. 5%, *p* = 0.61). Although tumor involvement of OCD is a reported risk factor for cholecystitis,^8^ this was present in only one of the three patients who developed cholecystitis in the EGIS group. As the bile duct diameters of the two patients in the EGIS group were smaller (5.3 and 7.2 mm, respectively) than that of the deployed SEMS, we speculate that bile outflow was impaired due to SEMSs compressing the OCD. The flared end of the EGIS stent might have also had an impact on the development of cholecystitis as the flared end was located near the OCD in these two patients. Bleeding due to pseudoaneurysm occurred in one patient in the EGIS group. As the pseudoaneurysm was located near the center of the stent and apart from the flared end, we believe that the flare structure of the stent was not associated with the development of the pseudoaneurysm.

There are several limitations in this study. First, this was a single‐center retrospective study with a limited sample size. Although we included consecutive patients with pancreatic cancer who underwent initial CSEMS placement, selection bias is inevitable. Second, differences in stent length may have affected the results of this study.

In conclusion, the EGIS biliary stent was not associated with longer TRBO compared to c‐CSEMS. Further improvements, especially against stent migration, are needed to improve its efficacy.

## CONFLICT OF INTEREST STATEMENT

Tsuyoshi Takeda received honoraria from Boston Scientific Japan and SB‐Kawasumi Laboratories. Takashi Sasaki received honoraria from Boston Scientific Japan, Century Medical, SB‐Kawasumi Laboratories, JAPAN LIFELINE, and KANEKA MEDIX. Naoki Sasahira received honoraria from SB‐Kawasumi Laboratories.

## ETHICS STATEMENT

Approval of the research protocol by an Institutional Reviewer Board: This study was conducted in accordance with a master protocol (2023‐GB‐077) for gastrointestinal, hepatic, and pancreatobiliary diseases, which was approved by the institutional review board of our institution.

## PATIENT CONSENT STATEMENT

Informed consent for this study was waived due to its retrospective design. The study was publicized on the hospital website, allowing patients to opt out of this study for any reason.
